# Intake of prebiotic fibers and the risk of laryngeal cancer: the *PrebiotiCa* study

**DOI:** 10.1007/s00394-022-03030-7

**Published:** 2022-11-06

**Authors:** Federica Turati, Federica Concina, Paola Bertuccio, Federica Fiori, Maria Parpinel, Martina Taborelli, Valentina Rosato, Werner Garavello, Eva Negri, Carlo La Vecchia

**Affiliations:** 1 Unit of Medical Statistics and Biometry, IRCCS National Cancer Institute of Milan, Milan, Italy; 2 Department of Clinical Sciences and Community Health, University of Milan, Via G. Celoria, 22, 20133 Milan, Italy; 3 Clinical Epidemiology and Public Health Research Unit, Institute for Maternal and Child Health-IRCCS “Burlo Garofolo”, Trieste, Italy; 4 Department of Public Health, Experimental and Forensic Medicine, University of Pavia, Pavia, Italy; 5 Department of Medicine, University of Udine, Udine, Italy; 6 Unit of Cancer Epidemiology, CRO Aviano National Cancer Institute, IRCCS, Aviano, Italy; 7 Department of Otorhinolaryngology, School of Medicine and Surgery, University of Milano- Bicocca, Monza, Italy; 8 Department of Medical and Surgical Sciences, University of Bologna, Bologna, Italy

**Keywords:** Laryngeal cancer, Fiber, Prebiotics, Diet, Prevention

## Abstract

**Purpose:**

To evaluate whether the intake of specific fibers with prebiotic activity, e.g., inulin-type fructans (ITFs), fructo-oligosaccharides (FOSs), and galacto-oligosaccharides (GOSs), is associated with laryngeal cancer risk.

**Methods:**

Within the *PrebiotiCa* study, we used data from a case–control study (Italy, 1992–2009) with 689 incident, histologically confirmed laryngeal cancer cases and 1605 controls. Six prebiotic molecules (ITFs, nystose [FOS], kestose [FOS], 1F-β-fructofuranosylnystose [FOS], raffinose [GOS] and stachyose [GOS]) were quantified in various foods via ad hoc conducted laboratory analyses. Subjects’ prebiotic fiber intake was calculated by multiplying food frequency questionnaire intake by the prebiotic content of each food item. The odds ratios (OR) of laryngeal cancer for prebiotic fiber intake were calculated using logistic regression models, including, among others, terms for tobacco, alcohol, and total energy intake.

**Results:**

The intakes of kestose, raffinose and stachyose were inversely associated with laryngeal cancer, with ORs for the highest versus the lowest quartile of 0.70 (95% confidence interval, CI 0.50–0.99) for kestose, 0.65 (95% CI 0.45–0.93) for raffinose and 0.61 (95% CI 0.45–0.83) for stachyose. ITFs, nystose and 1F-β-fructofuranosylnystose were not associated with laryngeal cancer risk. Current smokers and heavy drinkers with medium–low intakes of such prebiotic fibers had, respectively, an over 15-fold increased risk *versus* never smokers with medium–high intakes and a five to sevenfold increased risk *versus* never/moderate drinkers with medium–high intakes.

**Conclusion:**

Although disentangling the effects of the various components of fiber-rich foods is complex, our results support a favorable role of selected prebiotic fibers on laryngeal cancers risk.

## Introduction

Laryngeal cancer is a relatively common malignancy, with an estimated 184,615 new cases and almost 100,000 deaths globally from the disease in 2020 [1]; incidence rates are approximately fivefold higher in men than women [2]. Tobacco [3] and alcohol [4] are the most important risk factors for laryngeal cancer, with a multiplicative effect on risk [5]. Dietary factors have also been implicated, with red meat increasing the risk [6], and diets rich in fruit and non-starchy vegetables, and their related components, decreasing the risk [7–10].

Fiber intake has been favorably related with the risk of head and neck cancers, including laryngeal cancer [11]. In a pooled analysis within the International Head and Neck Cancer Epidemiology (INHANCE) consortium, the odds ratio (OR) of laryngeal cancer for the highest *versus* the lowest quintile of fiber intake was 0.66 (95% confidence interval, CI, 0.54–0.82), with significant trend of decreasing risk across quintiles [12]; the investigation was based on 9 studies and over 1500 cases of laryngeal cancer. Among prospective studies addressing the topic, one including 76 incident cases of laryngeal cancer found non-significant inverse associations with the intakes of total, insoluble and soluble fibers; one found a strong inverse association with total fiber intake in women, but not in men [13], and a further study conducted in women only found no relation, based, however, on 21 laryngeal cancer cases only [14].

To our knowledge, however, no information is available on the effect of specific fiber types with prebiotic activity, such as inulin-type fructans (ITFs), fructo-oligosaccharides (FOSs) and galacto-oligosaccharides (GOSs) [15]. Prebiotics are defined as “substrates selectively used by host microorganisms conferring health benefits” [16]. These food components, which are mainly fibers, are resistant to hydrolysis by digestive enzymes and bypass digestion in the small intestine to the colon, where they are metabolized by the microbiota. Their consumption, therefore, modulates the gut microbiota composition [17, 18]. Noteworthy, dietary interventions involving mainly FOSs stimulate the growth of Bifidobacteria e Lactobacilli [19].

The *PrebiotiCa* (The role of prebiotics in the prevention of cancer, an integrated network of Italian case–control studies) project aims to assess the association between the intake of fibers with prebiotic activity and the risk of several common cancers using detailed dietary information collected through a valid [20] and reproducible [21, 22] food frequency questionnaire (FFQ). To address the issue, the amount of 6 prebiotic molecules (i.e., ITFs, nystose (FOS), kestose (FOS), 1F-β-fructofuranosylnystose (FOS), raffinose (GOS), and stachyose (GOS)) in several food products was estimated in ad hoc conducted laboratory analyses [23].

In the present investigation within the *PrebiotiCa* study, we evaluated the association between the intake of prebiotic fibers and the risk of laryngeal cancer.

## Methods

### Study design and data collection

Data for the current investigation derived from a case–control study on laryngeal cancer conducted between 1991 and 2009 in the provinces of Milan and Pordenone, northern Italy [24]. The study included 689 incident, histologically confirmed laryngeal cancer patients (620 men and 69 women; median age 62 years, range 21–80 years), admitted to major teaching and general hospitals of the study areas. Controls were 1605 subjects (1264 men and 341 women, median age: 62 years, range: 27–84) admitted to the same network of hospitals of cases for a wide spectrum of acute, nonneoplastic conditions unrelated to alcohol drinking, tobacco use, or long-term dietary modifications. To account for the rarity of laryngeal cancer in women, a control-to-case ratio of about 5 was chosen for women, as opposed to about 2 for men. Less than 5% of cases and controls approached for interview declined study participation. The study was performed in line with the principles of the Declaration of Helsinki. All participants gave informed consent to participate. The study protocol was submitted to the Board of Ethics of the participating hospitals and received the approval required at the time of data collection. The Ethics Committees of the Hospital “Niguarda Ca’ Granda”, Milan, and of the National Cancer Institute “Centro di Riferimento Oncologico, IRCCS”, Aviano, provided the study approval (respectively, 1125/194 and IRB-15-2012).

Cases and controls were interviewed by centrally trained interviewers using a structured questionnaire, which included personal and sociodemographic characteristics, anthropometric measures, and lifestyle habits (smoking habits, alcohol drinking, and physical activity). Subjects’ usual diet in the 2 years preceding diagnosis (for cases) or hospital admission (for controls) was assessed using an interviewer-administered FFQ, which collected the average weekly consumption of 78 foods, food groups or complex recipes. A separate section collected history of consumption of alcoholic beverages. Intakes lower than once per week, but at least once per month, were coded as 0.5 per week. The FFQ was tested for reproducibility [21, 22] and validity [20]. In the validation study, the correlation coefficient between the intakes estimated from the FFQ and from two 7-day diaries was 0.58 of fibers, and around 0.60–0.65 for energy, available carbohydrates, sugar and starch, and around 0.50 for total, animal and vegetable proteins, and for animal fats and saturated fatty acids. As for reproducibility, correlation coefficients between intakes estimated by two FFQs were 0.67 for fiber, and between 0.6 and 0.7 for most of the FFQ items in the “bread, cereals and first courses” category, between 0.5 and 0.6 for most of the vegetables, root vegetables, tubers roots and legumes, and around 0.6–0.7 for various fruits.

### Quantification of prebiotic fibers in foods

The methodology used for the quantification of prebiotic fibers in foods was described in details [23]. In brief, FOSs (i.e., nystose, kestose and 1F-β-fructofuranosylnystose) and GOSs (raffinose and stachyose) were determined in 78 food sources; ITFs in 7. Food sampling and analysis were conducted at Neotron SPA in Modena, which has a certified laboratory for food analysis. The food products investigated included 15 types of fruits, 32 varieties of vegetables, root vegetables and tubers, 9 types of dried or fresh legumes, and 22 cereals and cereals-based products (both whole-grain and refined products), most of which assessed in the FFQ used in the present case–control study (as a specific FFQ item, as a food of an item including mixed foods, or as a food ingredient of an item consisting in complex recipes). The 78 samples (unique sample) analyzed in this study were collected from supermarkets located in Modena from 17 May to 24 June 2021.

ITFs were determinate using an internal analytical method based on AOAC 997.08 procedure. Freeze-dried samples were extracted in hot water (T equal to 85 °C) with an immediately pH check (pH equal to 6.5–8.0) and mild agitation (extract A_0_). A portion of extracted A_0_ was firstly hydrolyzed with a sufficient amount of amyloglucosidase solution, taking into account amount of starch and maltodextrins present (extract A_1_), and secondly hydrolyzed with a sufficient amount of inulinase solution, taking into account amount of fructans present, and enzyme concentration (Fructozyme) (extract A_2_). Extract A_0_, A_1_ and A_2_ were injected into a high-performance anion-exchange chromatography coupled to pulsed amperometric detection (HPAE-PAD), previous addition of 2.0 g of glucoheptose internal standard solution to determine the following sugars: glucose, fructose, sucrose, maltitol and galactose, and then calculate ITFs content using a specific formula. ITFs were determined in fresh onion, garlic, banana, leek, Jerusalem artichoke, artichoke and shallot. The analysis was performed based on a limit of detection (LOD) of the methodology equal to less than 0.005. ITFs content ranged from 25.1 g/100 g in garlic to 1 g/100 g in onion and leek [23].

FOSs and GOSs in fresh samples were determined according to Manali Aggrawal and Jeff Rohrer method (Thermo Scientific, Application Note 1149: profiling Fructosyloligosaccharides (FOS)-containing samples by HPAE-PAD. Sunnyvale, CA, 2015). One gram of homogenized sample was extracted with 200 mL of sodium hydroxide 0.0025 M and then analyzed using HPAE-PAD method. The LOD was between < 0.002 and < 0.010. The following molecules were quantified: raffinose (GOS), stachyose (GOS), nystose (FOS), kestose (FOS) and 1f-fructofuranosylnystose (FOS). Total FOSs was calculated as the sum of nystose, kestose and 1F-β-fructofuranosylnystose.

The principal source of FOS was Jerusalem artichoke (4.45 g/100 g) (not included in the FFQ), with other foods containing less than 1 g/100 g, and was represented principally as kestose. The primary source of GOSs were pulses, excluded green beans, with a mean content of 1.17 ± 0.87 g/100 g. In particular, raffinose was particularly abundant in dried peas (0.498 g/100 g) and chickpeas (0.463 g/100 g) and stachyose in dried beans (1.905 g/100 g) and peas (1.814 g/100 g) [23].

### Prebiotic and nutrient intake

The average daily total energy and nutrient intake was calculated by multiplying the reported frequency of consumption of food items by standard portion size and nutrient content per 100 g based on an Italian food composition database [25, 26]; for FOSs, GOSs and ITFs intake, we used laboratory data obtained as previously described.

### Statistical analysis

The ORs and the corresponding 95% CIs of laryngeal cancer according to quartiles of intake (based on the distribution among controls) of the selected prebiotic fibers were calculated using multiple unconditional logistic regression models. The models included terms for sex, age (quinquennia), study center, year of interview (continuous), years of education (< 7, 7–11, ≥ 12), alcohol intake (0–13, 14–27, 28–55, ≥ 56 drinks/week), tobacco smoking (never, ex, current smokers of < 15, 15–24, ≥ 25 cigarettes/day), body mass index (BMI, < 20, 20−  < 25, 25- < 30, ≥ 30 kg/m^2^) and non-alcohol energy intake (in quartiles, based on the distribution among controls). Tests for trends across quartiles were performed by including the examined variable as ordinal.

For prebiotic fibers showing significant associations in the main analysis, we conducted stratified analyses by age, education, years of education, BMI, tobacco smoking, and alcohol drinking, and evaluated their combined effects with alcohol drinking and tobacco use. As for subgroup analyses, heterogeneity across strata was tested using likelihood ratio tests.

All the analyses were conducted using the SAS software, version 9.4 (SAS Institute, Inc., Cary, NC, USA).

## Results

Table 1 gives the distribution of laryngeal cancer cases and controls according to selected factors. Cases were more frequently tobacco smokers and alcohol drinkers, and tended to report lower education and BMI.

**Table 1:** Distribution of 689 laryngeal cancer cases and 1605 controls by selected characteristics

	Cases	Controls
	*N* (%)	*N* (%)
Age		
< 50	71 (10.3)	154 (9.6)
50–59	195 (28.3)	471 (29.4)
60–69	278 (40.4)	658 (41.0)
≥ 70	145 (21.0)	322 (20.1)
Sex		
Men	620 (90.0)	1264 (78.8)
Women	69 (10.0)	341 (21.2)
Period of interview		
1991–1994	160 (23.2)	441 (27.5)
1995–1999	276 (40.1)	585 (36.5)
2000–2004	168 (24.4)	327 (20.4)
2005–2009	85 (12.3)	252 (15.7)
Education (years)^a^		
< 7	402 (58.4)	833 (51.9)
7–11	185 (26.9)	488 (30.4)
≥ 12	100 (14.5)	279 (17.4)
Tobacco smoking^a^		
Never smoker	44 (6.4)	603 (37.6)
Ex smoker	240 (34.8)	608 (37.9)
Current smoker (cigarettes/day)		
< 15	67 (9.7)	164 (10.2)
15–24	214 (31.1)	162 (10.1)
≥ 25	120 (17.4)	65 (4.1)
Alcohol drinking (drinks/week)^a^		
0–13	120 (17.4)	561 (35.0)
14–27	140 (20.3)	488 (30.4)
28–55	206 (29.9)	381 (23.7)
≥ 56	223 (32.4)	172 (10.7)
Body mass index (kg/m^2^)^a^		
< 20	39 (5.7)	52 (3.2)
20–24.99	285 (41.4)	572 (35.6)
25–29.99	281 (40.8)	761 (47.4)
≥ 30	80 (11.6)	218 (13.6)

Italy, 1991–2009

^a^the sum does not add up to the total because of missing values

Among control subjects, median intakes (mg/day) were 774 for ITFs, 169 for kestose, 16 for nystose, 2 for 1F-β-fructofuranosylnystose, 95 for raffinose, and 185 for stachyose. Kestose intake accounted for 90% of total FOSs intake; nystose for 8% and 1F-β-fructofuranosylnystose for 2%.

Table 2 provides the ORs of laryngeal cancer according to prebiotic fiber intake. Kestose was inversely associated with laryngeal cancer, with an OR of 0.70 (95% CI 0.50–0.99) for the highest versus the lowest quartile of intake (p for trend across quartiles: 0.06). There was no association with the other members of the FOSs family as well as with ITFs. As for GOSs, the ORs for the highest versus the lowest quartile of intake were 0.65 (95% CI 0.45–0.93, p for trend across quartiles: 0.004) for raffinose and 0.61 (95% CI 0.45–0.83, p for trend: 0.001) for stachyose.

**Table 2:** Odds ratios (OR) of laryngeal cancer and corresponding 95% confidence intervals (CI) according to prebiotic fiber intake

	Quartiles^a^				*P*_trend_
	Q1	Q2	Q3	Q4	
Inulin fraction (mg)					
Upper cutpoint	407	774	1460	–	
Cases (%)	191 (27.7)	152 (22.1)	152 (22.1)	194 (28.2)	
OR^b^ (95% CI)	1^c^	0.74 (0.55–0.99)	0.65 (0.48–0.88)	0.87 (0.65–1.17)	0.273
Kestose (FOS) (mg)					
Upper cutpoint	133	169	218		
Cases (%)	180 (26.1)	171 (24.8)	158 (22.9)	180 (26.1)	
OR^b^ (95% CI)	1^c^	0.74 (0.54–1.00)	0.71 (0.51–0.98)	0.70 (0.50–0.99)	0.060
Nystose (FOS) (mg)					
Upper cutpoint	12	16	20	–	
Cases (%)	123 (17.9)	165 (23.9)	199 (28.9)	202 (29.3)	
OR^b^ (95% CI)	1^c^	1.16 (0.84–1.59)	1.18 (0.85–1.64)	0.96 (0.67–1.37)	0.734
1F-β-fructofuranosylnystose (FOS) (mg)					
Upper cutpoint	0.7	1.9	6.9	–	
Cases (%)	213 (30.9)	178 (25.8)	143 (20.8)	155 (22.5)	
OR^b^ (95% CI)	1^c^	0.96 (0.72–1.27)	0.69 (0.51–0.93)	0.74 (0.55–1.01)	0.013
Total FOS (mg)					
Upper cutpoint	150	190	242		
Cases (%)	170 (24.7)	176 (25.5)	163 (23.7)	180 (26.1)	
OR^b^ (95% CI)	1^c^	0.81 (0.59–1.10)	0.78 (0.56–1.09)	0.73 (0.51–1.04)	0.100
Raffinose (GOS) (mg)					
Upper cutpoint	76	95	116	–	
Cases (%)	171 (24.8)	196 (28.4)	154 (22.4)	168 (24.4)	
OR^b^ (95% CI)	1^c^	0.99 (0.73–1.33)	0.68 (0.49–0.95)	0.65 (0.45–0.93)	0.004
Stachyose (GOS) (mg)					
Upper cutpoint	112	185	301	–	
Cases (%)	182 (26.4)	191 (27.7)	169 (24.5)	147 (21.3)	
OR^b^ (95% CI)	1^c^	0.93 (0.70–1.23)	0.81 (0.60–1.09)	0.61 (0.45–0.83)	0.001

Italy, 1991–2009

*FOS* fructo-oligosaccharides, *GOS* galacto-oligosaccharides

^a^derived among controls

^b^estimates from logistic regression models adjusted for age, sex, center, year of interview, years of education, tobacco smoking, alcohol intake, body mass index and non-alcohol energy intake

^c^reference category

In subgroup analyses (Table 3), no significant heterogeneity was observed in strata of age, education, BMI, tobacco smoking and alcohol drinking, although the ORs comparing the highest versus the lowest quartile of intake were no longer significant in some strata.

**Table 3:** Odds ratios (OR) of laryngeal cancer for the highest *versus* the lowest quartile of kestose, raffinose, and stachyose intake across strata of selected covariates

	Kestose (FOS)	Raffinose (GOS)	Stachyose (GOS)
	OR^a^ (95% CI)	OR^a^ (95% CI)	OR^a^ (95% CI)
Age			
< 62	0.82 (0.49–1.37)	0.89 (0.52–1.51)	0.47 (0.29–0.75)
≥ 62	0.64 (0.39–1.03)	0.50 (0.30–0.83)	0.74 (0.49–1.12)
* p*_*heterogeneity*_^*b*^	0.125	0.331	0.321
Education (years)			
< 7	0.60 (0.38–0.97)	0.61 (0.37–0.99)	0.65 (0.43–0.99)
≥ 7	0.81 (0.48–1.37)	0.69 (0.40–1.20)	0.59 (0.37–0.93)
* p*_*heterogeneity*_^*b*^	0.716	0.638	0.474
Body mass index (kg/m^2^)			
< 25	0.71 (0.42–1.21)	0.50 (0.29–0.88)	0.54 (0.34–0.85)
≥ 25	0.69 (0.43–1.10)	0.75 (0.46–1.22)	0.69 (0.45–1.05)
* p*_*heterogeneity*_^*b*^	0.469	0.335	0.526
Tobacco smoking			
Ex smoker	0.66 (0.39–1.10)	0.64 (0.37–1.09)	0.68 (0.43–1.07)
Current smoker	0.64 (0.38–1.06)	0.76 (0.45–1.30)	0.58 (0.37–0.91)
* p*_*heterogeneity*_^*b*^	0.664	0.634	0.804
Alcohol drinking (drinks/week)			
0–27	0.65 (0.39–1.11)	0.94 (0.54–1.62)	0.73 (0.47–1.14)
≥ 28	0.74 (0.47–1.18)	0.50 (0.30–0.81)	0.56 (0.36–0.85)
* p*_*heterogeneity*_^*b*^	0.745	0.353	0.339

Italy, 1991–2009

*CI* confidence interval, *FOS* fructo-oligosaccharides, *GOS* galacto-oligosaccharides

^a^estimates from logistic regression models adjusted for age, sex, center, year of interview, years of education, tobacco smoking, alcohol intake, body mass index, and non-alcohol energy intake, unless the variable was the stratification factor

^b^the test for heterogeneity considered all four quartiles of intake

**Fig. 1:**
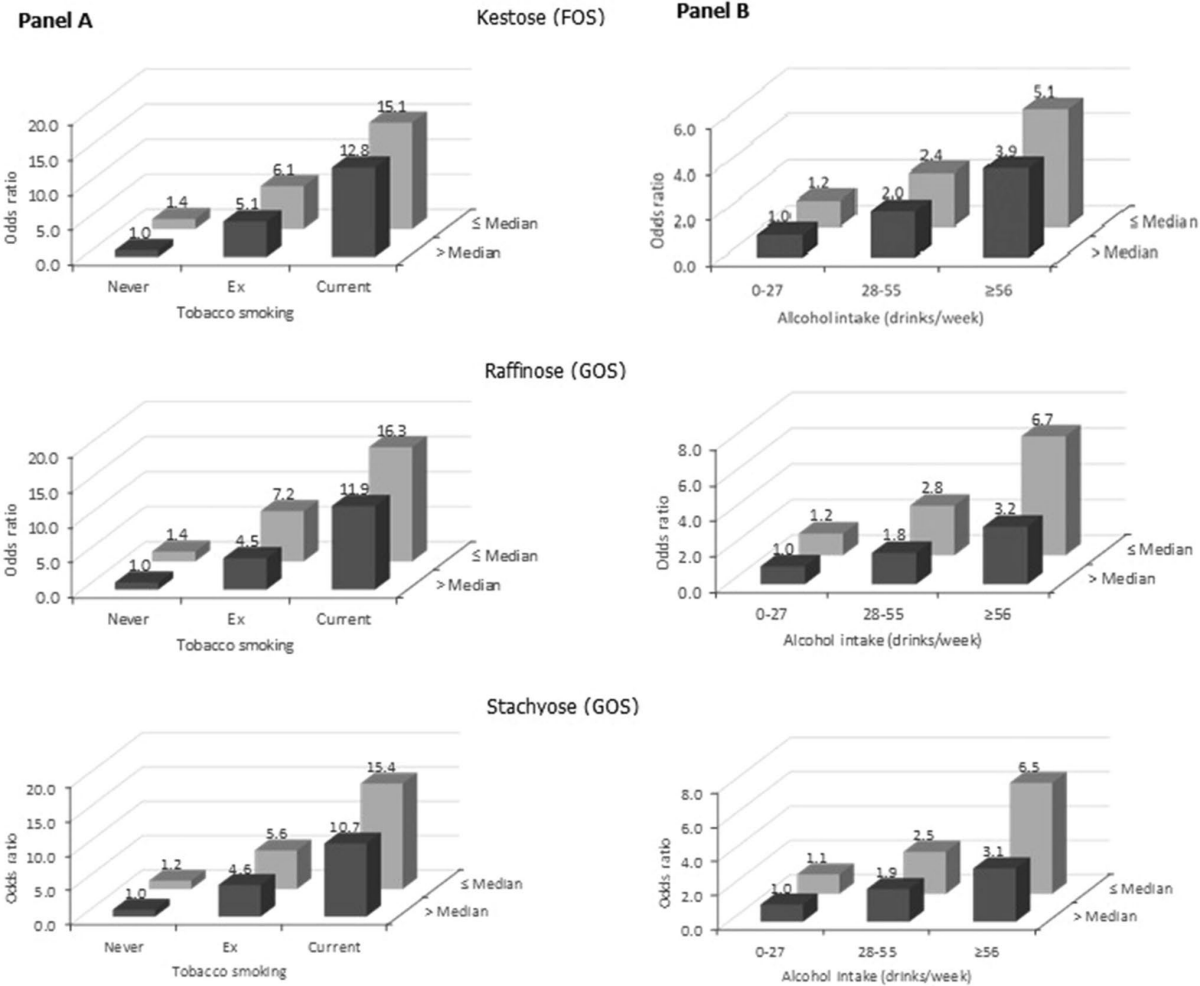
Odds ratios^a^ (OR) of laryngeal cancer for combined categories of prebiotic fiber intake and tobacco smoking (Panel **A**) or alcohol intake (Panel **B**). Italy, 1991–2009. *FOS* fructo-oligosaccharides, *GOS* galacto-oligosaccharides. ^a^Estimates from logistic regression models adjusted for age, sex, center, year of interview, years of education, body mass index, and non-alcohol energy intake. The ORs for combined categories of tobacco smoking and prebiotic fiber intake were additionally adjusted for alcohol intake; the ORs for combined categories of alcohol and prebiotic fiber intake were additionally adjusted for tobacco smoking

Figure 1 shows the combined effect of prebiotic fiber intake and tobacco smoking (Panel A) or alcohol intake (Panel B) on laryngeal cancer risk. Compared to never smokers with prebiotic fiber intake above the median value, the OR for current smokers with a lower prebiotic fiber intake was above 15 for the three investigated prebiotic fibers. When we splitted current smokers into two categories based on the amount of cigarettes smoked, compared to the same low-risk category, the OR for smokers of ≥ 15 cigarettes/day with prebiotic fiber intake below the median value was around 36–37 for kestose and raffinose, and about 31 for stachyose (data not shown). As for the combined effect with alcohol, compared to never/moderate alcohol drinkers with prebiotic fiber intake above the median value, the OR for heavy drinkers with a lower intake of prebiotic fiber was about 5 for kestose and about 6.5 for raffinose and stachyose.

## Discussion

In this large, multicentric, Italian study the intake of selected prebiotic fibers, namely the two members of the GOSs family raffinose and stachyose and the FOS kestose, were inversely associated with laryngeal cancer. Tobacco use and alcohol drinking are the strongest risk factor for the disease. We found that, compared to never smokers with medium–high intake of such prebiotic fibers, current smokers with lower intakes had a ~ 15-fold increased laryngeal cancer risk. In addition, compared with never/moderate drinkers with medium–high intake of the prebiotic fibers, heavy alcohol drinkers with lower intakes had a 5- to sevenfold increased risk.

Fibers may lower glycemic response, improve insulin sensitivity, and reduce insulin-like growth factors (IGF-1), which is a promoter of carcinogenesis at various sites [27]. Fibers with prebiotic activity are selectively fermented by specific-health promoting colonic bacteria; the major by-products of the bacterial fermentation are short chain fatty acids (SCFAs), primarily acetate, propionate, and butyrate, which have potent anti-neoplastic properties [28, 29]. In any case, evidence on the favorable role of SCFAs in cancer occurrence and progression mainly, but not exclusively, focuses on colorectal cancer.

Based on a partially overlapping set of laryngeal cases and controls with the present analysis, we previously reported a strong inverse association between fiber intake (total, soluble, insoluble, from vegetables and from fruit) and laryngeal cancer risk [30]. While other studies addressed the topic [12–14, 31], no previous one has focused on specific fibers with prebiotic activity. In a parallel analysis within the same *PrebiotiCa* study, we found that higher raffinose and stachyose intakes were inversely associated with colorectal cancer, while the intake of ITFs and FOSs did not influence the risk [32].

Of the foods investigated in laboratory analyses and considered in the FFQ, legumes (peas, chickpeas and beans), wholemeal flour, whole-grain based products, and barley had the highest contents of GOSs [23]. Raffinose was detected in certain amounts also in white wheat flour and wheat products, while stachyose was detected almost exclusively in legumes (beans, followed by peas, chickpeas and lentils). As for FOSs, kestose was abundant in shallot, garlic, whole wheat pasta, wholemeal biscuits, banana and barley; legumes had low or undetectable kestose concentrations. Nystose, and in particular 1F-β-fructofuranosylnystose, were detected in very small concentrations in a limited range of foods (e.g., shallot, garlic and barley), consumed in small amounts by our population. As such, low daily intakes of nystose and 1F-β-fructofuranosylnystose were estimated in our database, with limited variation across subjects. Only 6 foods in our FFQ were significant sources of ITFs. ITFs were very abundant in garlic, but when amount consumed was considered, bananas were the most important source, accounting alone for over 60% of ITFs intake in our population. Again, accounting for amount consumed, cereal-based products and legumes were the largest contributors to raffinose and stachyose intakes in our population, while other food groups, including vegetables and fruit, provided limited contributions; kestose mainly came from cereals and fruit in similar proportions.

Legume consumption has been favorably associated with the risk of head and neck cancer [33], including laryngeal cancer [34, 35]; in addition, fiber from legumes inversely related to head and neck cancer, in men only, in one study [13]. Data on cereals and grains are very scanty; whole and refined grains have been inversely related with head and neck cancer in women in one study [13], while a pooled analysis conducted within the INHANCE consortium showed no association with cereals and grains (head and neck cancer) [7].

Our study is retrospective and hospital-based. However, selection bias should be limited as we excluded from the control group patients admitted to hospital for chronic conditions, digestive tract diseases, or diseases associated with alcohol drinking, tobacco use, or long-term dietary modifications; participation of cases and controls was satisfactory (> 95%); and cases and controls were identified in the major teaching and general hospitals of the areas under surveillance. The similar interview setting for cases and controls weights against information bias, and, although recall bias is possible, this should not be different based on the disease status. In addition, the FFQ gave satisfactory results when tested for validity [20] and reproducibility [21, 22] of food intakes.

The study was conducted over a relatively long period, in which dietary habits of study subjects might have changed. However, any change should have occurred similarly in cases and controls, and hence not have influenced appreciably our results. Furthermore, our risk estimates were adjusted for year of interview.

As for possible confounding, we were able to adjust for major risk factors for the neoplasm as well as for total energy intake; still, residual confounding cannot be ruled out. Prebiotics are types of fibers, and adjusting for total fiber intake is, therefore, an over adjustment. When this was made (data not shown), the associations observed with the two GOSs and with kestose were largely attenuated. In any case, ORs not adjusted for total fiber intake are more valid estimates of the association.

Investigation of the association between intake of prebiotic fiber and disease risk is limited by challenges in estimating individual intakes of prebiotic fibers from questionnaires data, lack of published food composition data and heterogeneity in methodologies. Furthermore, the definition of ITFs is not satisfactorily agreed upon. Our FFQ was not specifically designed to assess the intake of prebiotic fibers and it did not include items on specific dietary products reported to contain prebiotic fibers, including rye products, spelt, Jerusalem artichoke, breakfast cereal products, oats and soya beans, nor it distinguished whole-grain from non-whole-grain items, apart from bread. However, consumption of these foods and whole-grain cereals was uncommon in our population at the time of data collection, and hence their contribution to subjects’ daily prebiotic intake and to the prebiotic—laryngeal cancer association was likely to be minimal; further, open questions allowed to collect data on foods/recipes not included as FFQ item. In any case, any possible misclassification of individual estimates of prebiotic fiber intake is unlikely to be unbalanced between cases and controls, and hence unlikely to lead to an overestimation of the association.

Another limitation relates to the application of results from food content analyses conducted in 2021 to dietary intakes collected in the 1990s’ and 2000’s, since the contents of ITFs, FOSs and GOSs in food sources might have changed. However, at the time of study conduction, there was a lack of comprehensive food composition data regarding these prebiotics in commonly consumed foods, with no prior data for Italian food sources. The few available data were scattered across studies conducted outside Europe which applied heterogeneous methodologies for the quantification of prebiotic molecules, and showed wide variation in prebiotic food composition [23]. Further, the application of food composition data to dietary data collected at a different time point, when contemporary data are not available, is a common approach in nutritional studies [36].

In conclusion, the present data are suggestive of a favorable role of selected prebiotic fibers, in particular those of the GOSs family (i.e., raffinose and stachyose), on laryngeal cancers risk. However, other components of fiber-rich foods may also influence laryngeal cancer risk. Disentangling the effects of various food components is extremely difficult, and hence caution in inference is required.
